# 104 Parkin-independent Pathway of Mitophagy as a Potential Target Mitochondrial Dysfunctions in Burns

**DOI:** 10.1093/jbcr/irac012.107

**Published:** 2022-03-23

**Authors:** Jingyuan Chen, Yoh Sugawara, Hiroyuki Morinaga, Jeevendra Martyn, Shingo Yasuhara

**Affiliations:** The First Affiliated Hospital of Sun Yat-Sen University, Guangzhou, Guangdong; Shriners Hospital for Dallas, Boston, Boston, Massachusetts; Kyorin University, Tokyo, Tokyo; Shriners Hospitals for Dallas, Boston, Massachusetts; Shriners Hospital for Dallas, Boston, Massachusetts

## Abstract

**Introduction:**

In many critical illnesses including burn injury (BI), muscle wasting (MW) with mitochondrial dysfunction (MD) leads to poor prognosis. Disturbed mitochondria can normally be turned over by autophagic degradation of mitochondria (mitophagy). We have previously observed that BI causes disturbed mitophagy response in skeletal muscles both in vivo and in the cultured myocytes, a potential mechanism for BI-induced MD. These previous findings have lead to the expectation that augmenting mitophagy will rescue mitochondrial functions and can help treating the BI-induced MW and MD. There have been, however, limited research tools to specifically intervene (or augment) mitophagy. In the current study, we have established mitophagy-compromised cell strains by CRISPR/Cas9-mediated knocking out of BNIP3L, a pivotal molecule mediating parkin-independent mitophagy induction. Deferiprone (DFP), a recently established mitophagy inducer, had previously been known to exert cellular and organ protective functions, but the mechanisms of its beneficial effects were not investigated in detail. Nor has its efficacy been tested on BI-induced MD.

**Methods:**

First, whether DFP stimulated mitophagy induction causes translocation of mitophagy markers into mitochondrial fraction, was tested by Western Blotting on the WT C2C12 cells against Parkin/PINK1- and BNIP3L- pathway molecules. Next we established BNIP3L knockout C2C12 myoblast cell like by CRISPR/Cas9-mediated gene deletion. Using both WT and BNIP3L knockout (KO) cell lines incubated in the BI or SB serum (from 30% rat burn at 3PBD) with or without DFP treatment, we tested whether DFP can rescue BI-induced upregulation of mitochondria-derived superoxide (SO) using MitoSOX staining.

**Results:**

In WT C2C12 cells, mitophagy stimulation by DFP caused robust increase of the protein amount of BNIP3L in the mitochondrial fraction, but Parkin/PINK1 did not. KO completely abolished both basal and stimulated increase of BNIP3L. BI serum caused significant elevation of mitochondrial SO in WT myocytes ( >20 fold of SB), which was ameliorated by DFP-stimulated mitophagy augmentation (95% reduction). In KO C2C12 cells, however, DFP-induced SO reduction in BI serum was completely abolished, suggesting that DFP-mediated mitochondrial protection against BI was through augmenting mitophagy via BNIP3L pathway.

**Conclusions:**

By using BNIP3L KO C2C12 myocytes, mitochondrial protective role of BNIP3L-mediated mitophagy against BI-induced stress was demonstrated for the first time. Previously reported cellular and organ protective functions of DFP is likely through activation of this pathway. Augmentation of mitophagy will be a promising therapeutic approach in protecting BI-induced MW and MD.

